# Resistance Profiles of *Salmonella* Isolates Exposed to Stresses and the Expression of Small Non-coding RNAs

**DOI:** 10.3389/fmicb.2020.00130

**Published:** 2020-02-28

**Authors:** Huhu Wang, Mingyuan Huang, Xianming Zeng, Bing Peng, Xinglian Xu, Guanghong Zhou

**Affiliations:** ^1^Jiangsu Collaborative Innovation Center of Meat Production and Processing, Quality and Safety Control, Nanjing Agricultural University, Nanjing, China; ^2^College of Animal Medicine, Xinjiang Agricultural University, Ürümqi, China

**Keywords:** *Salmonella*, survival, acidity, hypertonicity, oxidation, non-coding small RNA

## Abstract

*Salmonella* can resist various stresses and survive during food processing, storage, and distribution, resulting in potential health risks to consumers. Therefore, evaluation of bacterial survival profiles under various environmental stresses is necessary. In this study, the resistance profiles of five *Salmonella* isolates [serotypes with Agona, Infantis, Typhimurium, Enteritidis, and a standard strain (ATCC 13076, Enteritidis serotype)] to acidic, hyperosmotic, and oxidative stresses were examined, and the relative expressions of non-coding small RNAs were also evaluated, including *CyaR*, *MicC*, *MicA*, *InvR*, *RybB*, and *DsrA*, induced by specific stresses. The results indicated that although all tested strains displayed a certain resistance to stresses, there was great diversity in stress resistance among the strains. According to the reduction numbers of cells exposed to stress for 3 h, *S.* Enteritidis showed the highest resistance to acidic and hyperosmotic stresses, whereas ATCC 13076 showed the greatest resistance to oxidative stress, with less than 0.1 Log CFU/ml of cell reduction. Greater resistance of cells to acidic, hyperosmotic, and oxidative stresses was observed within 1 h, after 2 h, and from 1 to 2 h, respectively. The relative expression of sRNAs depended on the isolate for each stress; acidic exposure for the tested isolates induced high expression levels of *DsrA*, *MicC*, *InvR*, *RybB*, *MicA*, and *CyaR*. The sRNA *RybB*, associated with sigma E and outer membrane protein in bacteria, showed a fold change of greater than 7 in *S.* Enteritidis exposed to the tested stresses. *CyaR* and *InvR* involved in general stress responses and stress adaptation were also induced to show high expression levels of *Salmonella* exposed to hyperosmotic stress. Overall, these findings demonstrated that the behaviors of *Salmonella* under specific stresses varied according to strain and were likely not related to other profiles. The finding also provided insights into the survival of *Salmonella* subjected to short-term stresses and for controlling *Salmonella* in the food industry.

## Introduction

Foodborne diseases, mainly caused by ingestion of water and food contaminated by microorganisms, have become a global health concern. Pathogens are responsible for millions of cases of foodborne illnesses every year; in the U.S. alone, 841 foodborne disease outbreaks were reported in 2017, resulting in 14,481 illnesses, 827 hospitalizations, and 14 food product recalls (Centers for Disease Control and Prevention [CDC], 2019). *Salmonella* can cause a wide range of human diseases, including gastroenteritis and septicemia, and is the leading cause of food poisoning associated with foodborne pathogens, resulting in millions of recalls and outbreaks. *Salmonella* has been identified in various foodstuffs and environments, particularly animal-derived food products, such as poultry and eggs (Carrasco et al., 2012). Microorganism contamination can occur at any point in the food production chain, from animal slaughter to food manufacturing and distribution. Many strategies and disinfectants, such as good hygiene and handling practices, chlorite-based sanitizers, and salt-cured processes, have been applied to avoid and/or reduce bacterial contamination in the food industry (Khan et al., 2017; Yoon and Lee, 2018); however, these harsh conditions actually represent serious stresses to microorganisms surviving in the corresponding surroundings. When encountering these stresses, most bacteria are rapidly injured and killed, whereas some of the cells survive by triggering adaptive cell responses to specific environmental stresses; when bacteria are not inactivated by environmental stresses, then they may adapt in response to various environmental challenges or stresses, and become more persistent strains. The tolerance response of pathogens to one or more stresses is referred to as stress resistance and has been shown to aid in the survival of pathogens in food products and in the food processing environment, and harm consumers. Besides the numbers of survival cells, the intracellular profiles were also altered by the stresses (Begley and Hill, 2015; Wang et al., 2016; Pradhan and Negi, 2019).

Acidity, oxidation, and hypertonicity are stresses that are commonly encountered by *Salmonella* strains in the food industry. Food-contacted surfaces disinfected by hydrogen peroxide or sodium hypochlorite become oxidative and acidic environments. Some foods, such as juice and seafood, provide acidic and hyperosmotic environments for *Salmonella* to survive. For bacteria existing in foods or related environments, cellular tolerance to stress is widely observed, and the reduction numbers of bacteria subjected to a certain stress were commonly applied to evaluate the primary resistance profiles. Although many studies have focused on the stress response, the factors studied have varied greatly to include growth dynamics, morphology, and biofilm formation (Hazeleger et al., 2006; Boons et al., 2013). Moreover, strains within a given species usually display various resistance in terms of their general physiology and ability to respond to specific stresses, and the isolate-dependent stress responses for *Salmonella* have not yet been examined.

When exposed to various stresses in a short-term period, bacteria can activate several survival mechanisms, ranging from surface structure modifications to the modulation of genes responsible for stresses (Esbelin et al., 2018; Orihuel et al., 2019). The repression of redundant protein expression and synthesis of necessary resources to counteract possible damage from the external environment are common gene modulation mechanisms (Hahm and Bhunia, 2006). Many studies have concentrated on the fundamental aspects of bacterial survival, including cell membrane modification, DNA modification, transcriptional and translational responses, trigger factors, and initiation factors (Begley and Hill, 2015; Burgess et al., 2016). The molecular bases for stress response have also been explored, particularly for special stress-induced protein synthesis, such as glutamate decarboxylase in acid stress and superoxide dismutase in oxidative stress. These stress-induced proteins may result in increased intracellular pH, protection or repair of proteins and DNA, and induction of modifications to the membrane composition. In addition, several central regulators of general stress responses, such as RNA polymerase and sigma S (RpoS), have also been extensively characterized (Abdullah et al., 2017).

With the development of advanced sequencing methods for microorganisms, non-coding small RNAs (sRNAs), as post-transcriptional regulators ranging from 50 to 300 nucleotides in length, have been identified in the genomes of various microorganisms, such as *Salmonella* and *Listeria monocytogenes* (Koo et al., 2011; Kröger et al., 2012). These sRNAs have been shown to be involved in the regulation of bacterial growth, pathogenies, stress responses, and biofilm formation (Vogel, 2009; Hoe et al., 2013). Jin et al. (2009) demonstrated that the sRNA *GcvB* can upregulate *rpoS* levels to enhance the bacterial ability to survive in an acidic environment. Additionally, a low-temperature environment can induce the transcription of the sRNA *DsrA* and upregulate the expression of *rpoS* to promote survival (Mika and Hengge, 2014). Improving understanding of how changes in bacteria occur at the sRNA level may help to enhance knowledge of stress responses and design and develop more effective strategies to control *Salmonella* in order to ensure food safety. However, few studies have examined the behaviors of sRNAs when *Salmonella* are exposed to environmental stresses (Ryan et al., 2018; Barnhill et al., 2019).

In this study, the aim is to evaluate cellular responses to hyperosmotic, acidic, and oxidative stresses in several serotypes of *Salmonella* isolated from poultry meat and processing surfaces (*S.* Enteritidis, *S.* Agona, *S.* Infantis, and *S.* Typhimurium), and a standard strain *S.* ATCC 13076 with Enteritidis serotype was also tested as a reference isolate. Additionally, the changes in the expression of several sRNAs induced by stresses were examined. These findings are expected to provide insights into the mechanisms mediating the survival of *Salmonella* subjected to short-term stresses.

## Materials and Methods

### Bacterial Strains and Growth Conditions

The standard strain *Salmonella* ATCC 13076 with Enteritidis serotype and four strains isolated from poultry meat and meat processing surfaces, including *S.* Enteritidis, *S.* Agona, *S.* Infantis, and *S.* Typhimurium, were used in this study. Strains were stored at −80°C and cultured twice in trypticase soy broth (TSB; Hope-Biotechnology, Co., Ltd., Beijing, China) at 37°C for 20 h. The cells were harvested by centrifugation at 8000 × *g* for 5 min at 4°C and washed three times with 0.85% NaCl solution. Subsequently, the pellets were resuspended in 0.85% NaCl solution to a final concentration of approximately 10^9^CFU/L by measuring the OD value at 600 nm.

### Responses of *Salmonella* Isolates to Environmental Stresses

For acidic stress, hydrochloric acid was used to adjust the pH of TSB to 3.0; this was then used as acidified growth medium. For hyperosmotic stress, an appropriate amount of sodium chloride was added to TSB for a final concentration of 8.0% (w/v). Finally, oxidative medium was prepared by adding hydrogen peroxide to TSB for a final concentration of 2 mM to induce oxidative stress. TSB without any modifications was used as a control. For each stress and the control, 0.5 ml of each strain (approximately 10^9^ CFU/ml) was transferred to 4.5 ml of the appropriate TSB, and the samples were then incubated at 37°C for 1, 1.5, 2, or 3 h. After treatment, phosphate-buffered saline (pH 7.2) and sodium thiosulfate solution (w/v, 0.8%) were used to neutralize specific stresses. At various time points, the appropriate dilution of cultures was selected and plated manually on trypticase soy agar plates (Hope-Biotechnology, Co., Ltd.). After all the plates were cultured at 37°C for 24 h, the colonies were manually counted.

### Total RNA Isolation

Bacteria were exposed to various stresses for 3 h and collected. Cells without exposure to any stress were used as the control group. Total RNA in each group was isolated using an RNAiso plus Kit (Takara, Shiga, Japan) according to the manufacturer’s instructions, and each RNA was then treated with Takara recombinant DNase I. The RNA was finally reverse transcribed using a Takara RT master mix kit by incubation for 15 min at 37°C.

### Real-Time Polymerase Chain Reaction (PCR)

One microliter of cDNA was used as a template for real-time PCR with primers (Table 1). PCR was carried out with 10-μl reactions, prepared as follows: 5 μl of 2 × SYBR premix Ex SYBR, 0.2 μl of each primer (10 mM), 0.2 μl of ROX reference dye II, 1.0 μl of cDNA template, and 3.4 μl of RNase-free water. A QuantStudio 6 Flex system (Applied Biosystems, Foster City, CA, United States) was applied with the following protocol: initial denaturation at 95°C for 30 s; 40 cycles of denaturation at 95°C for 30 s and annealing at 60°C for 30 s; and a final melting curve program of 15 s at 95°C, 1 min at 60°C, and 15 s at 95°C. The relative expression of the tested sRNAs (including CyaR, MicC, InvR, RybB, MicA, and DsrA) in comparison with 16S DNA was analyzed using QuantStudio 6 Flex software from Applied Biosystems. The 2^–Δ^ ^Δ^ ^Ct^ method was used to determine the relative expression of each sRNA in comparison with the 16S rRNA gene as an internal control, where ΔΔCt = (C_t_, _sRNA_ − C_t_, _16__S__rR__NA_)_stress_ − (C_t_, _sRNA_ − C_t_, _16__S__rR__NA_)_control_. The relative fold change in the expression of genes was determined using five replicates. The relative levels of each sRNA were determined.

**TABLE 1:** Information of primers sequence.

**Target sRNA**	**Regulatory functions**	**Sequence of primers(5′–3′)**	**Length(nt)**	**References**
RybB	Associated with general stress responses and outer membrane protein	F: cgaaatggcggggttgatgg	78	Papenfort et al., 2006
		R: gccactgcttttctttgatgtcc		
MicC	Associated with general stress responses	F: acatccgttcgggcttgtca	109	Pfeiffer et al., 2009
		R: ttcattttgtcgctgggcca		
DsrA	Associated with survival and growth, and sigma E	F: ccctacgggtcgggatcaaa	87	Majdalani et al., 1998
		R: catcagatttcctggtgtaacgaattt		
CyaR	General stress responses (acidic, salt, nutrition, etc.)	F: acataacccataaatgctagctgtacc	86	Papenfort et al., 2008
		R: agggagattacgcaggccaa		
MicA	Involved in outer membrane protein and sigma E	F: cgcgcatttgttatcatcatccc	74	Papenfort et al., 2006
		R: ggagtggccaaaatttcatcgc		
InvR	Outer membrane protein involved in stress adaptation	F: acggttggccatttgtctctt	87	Pfeiffer et al., 2007
		R: gcgaggtgctgctgtatcaa		
16S DNA	Internal reference for qPCR test	F: aggccttcgggttgtaaagt R: gttagccggtgcttcttctg	96	Lee et al., 2009

### Statistical Analysis

Five replicates were evaluated for each experiment in this study (*n* = 5). Data for cell numbers were transformed to log 10 values of colony-forming units. Significant differences in cell numbers were evaluated using one-way Duncan’s analysis of variance, and differences with *p-*values of less than 0.05 were considered significant. All analyses were carried out using SPSS 18.0 software.

## Results

### Responses of Serotypes to Acidic Stress

**FIGURE 1:**
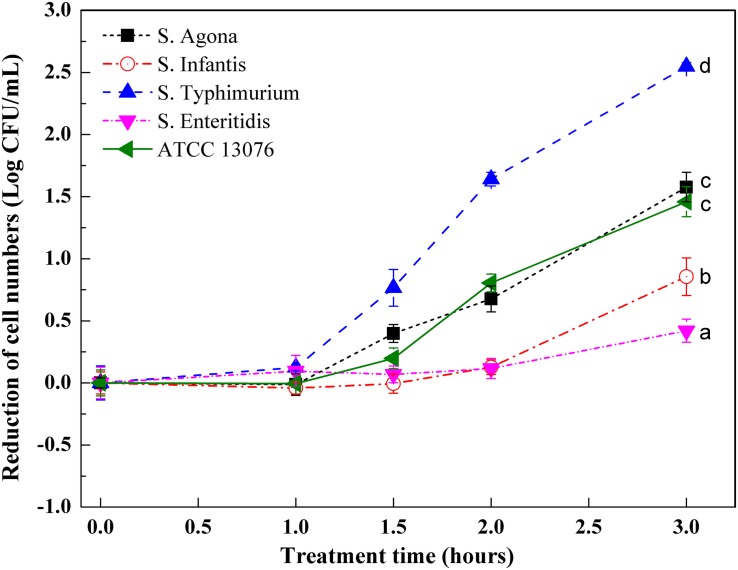
The variation of cell numbers of five *Salmonella* strains when exposed to acidic stress. Each symbol indicates the mean ± standard deviation of five independent experiments (*n* = 5). Different lowercase letters at treatment of 3 h indicated significant difference (*p* < 0.05).

Variations in *Salmonella* cell numbers in response to acidic stress are shown in Figure 1. All tested strains showed a profile of decreased cell numbers over time until 3 h, and no significant changes were observed in terms of cell numbers for *S.* Enteritidis. All strains showed steady counts of viable cells during the 1-h stress incubation period, and reduction of cell numbers then began to increase over the next 1 h. Dramatic reduction was observed from 1.5 to 2 h for ATCC 13076 and *S.* Typhimurium, with cell numbers showing reductions by 0.6 and 1.6 log CFU/ml, respectively. Compared with initial cell numbers, *S.* Typhimurium showed the largest decrease in cell number after exposure to acidic stress for 3 h, with about 2.5 log CFU/ml reduction. The various *Salmonella* strains showed decreasing resistance to acidic stress in the following order: *S.* Enteritidis > *S.* Infantis > *S.* ATCC 13076 > *S.* Agona > *S.* Typhimurium.

### Responses of Serotypes to Hyperosmotic Stress

**FIGURE 2:**
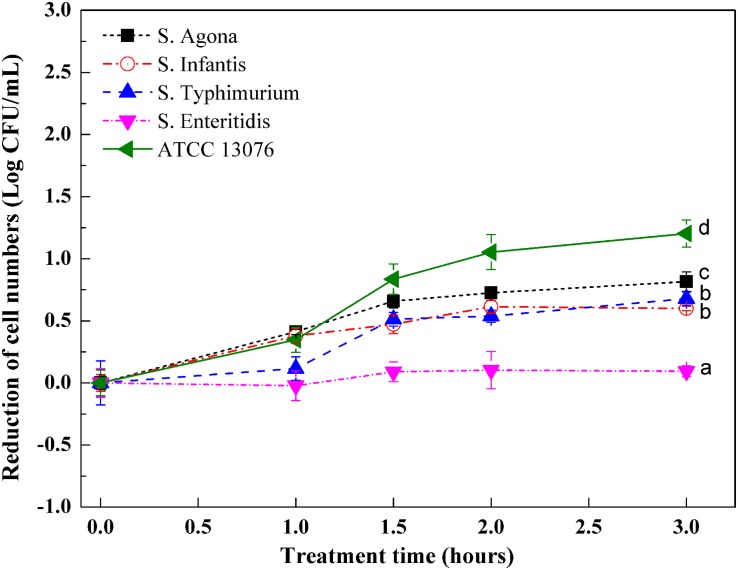
The variation of cell numbers of five *Salmonella* strains when exposed to hyperosmotic stress. Each symbol indicates the mean ± standard deviation of five independent experiments (*n* = 5). Different lowercase letters at treatment of 3 h indicated significant difference (*p* < 0.05).

Variations in the numbers of *Salmonella* exposed to hyperosmotic stress are shown in Figure 2. In general, except for *S.* Enteritidis, reduction of cell numbers for other tested strains increased significantly after exposure to stress for 3 h. During the first hour, the reduction of cell numbers for *S.* ATCC 13076, *S.* Agona, and *S.* Infantis began to increase, whereas those of *S.* Typhimurium and *S.* Enteritidis remained consistent. Major decreases were observed from 1 to 1.5 h, with *S.* ATCC 13076 showing the largest reduction of almost 0.8 log CFU/ml and *S.* Typhimurium showing a reduction of 0.5 log CFU/ml. However, this reduction trend slowed from 1.5 to 2 h. In the last hour, the cell numbers of all *Salmonella* strains remained steady. Among all *Salmonella* strains, great heterogeneity was observed after 3 h of exposure to hyperosmotic stress, with *S.* ATCC 13076 showing the largest reduction of 1.2 Log CFU/ml. During the entire stress period, *S.* Enteritidis showed the best ability to survive without major decreases in cell numbers. The various *Salmonella* strains showed decreasing resistance to hyperosmotic stress in the following order: *S.* Enteritidis > *S.* Typhimurium > *S.* Infantis > *S.* Agona > *S.* ATCC 13076.

### Responses of Serotypes to Oxidative Stress

**FIGURE 3:**
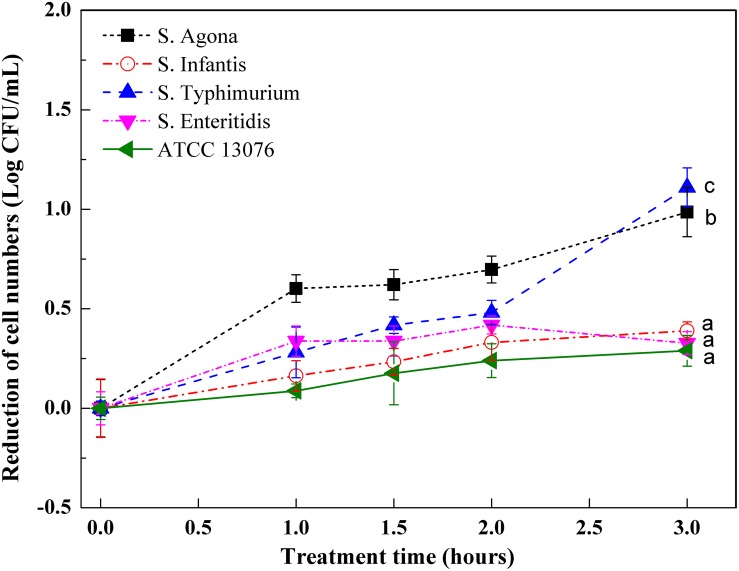
The variation of cell numbers of five *Salmonella* strains when exposed to oxidative stress. Each symbol indicates the mean ± standard deviation of five independent experiments (*n* = 5). Different lowercase letters at treatment of 3 h indicated significant difference (*p* < 0.05).

Figure 3 shows variations in *Salmonella* strains subjected to oxidative stress. Compared with hyperosmotic and acidic stresses, the tested strains showed less resistance to oxidative stress, with roughly 1 log reduction in cell numbers. All tested strains showed similar growth trends when exposed to an oxidative environment. For ATCC 13076, the numbers were almost steady throughout the 3-h incubation period. However, for the other strains, the cell numbers were significantly reduced within the first hour of incubation. As the incubation time increased to 3 h, the resistances of *S.* Typhimurium and *S.* Agona were found to be lower than those of the other three tested strains; the largest reduction was about 1 log CFU/ml. The various *Salmonella* strains showed decreasing resistance to oxidative stress in the following order: *S.* ATCC13076 > *S.* Enteritidis > *S.* Infantis > *S.* Agona > *S.* Typhimurium.

### Relative Expression of sRNAs in *Salmonella* Serotypes

**FIGURE 4:**
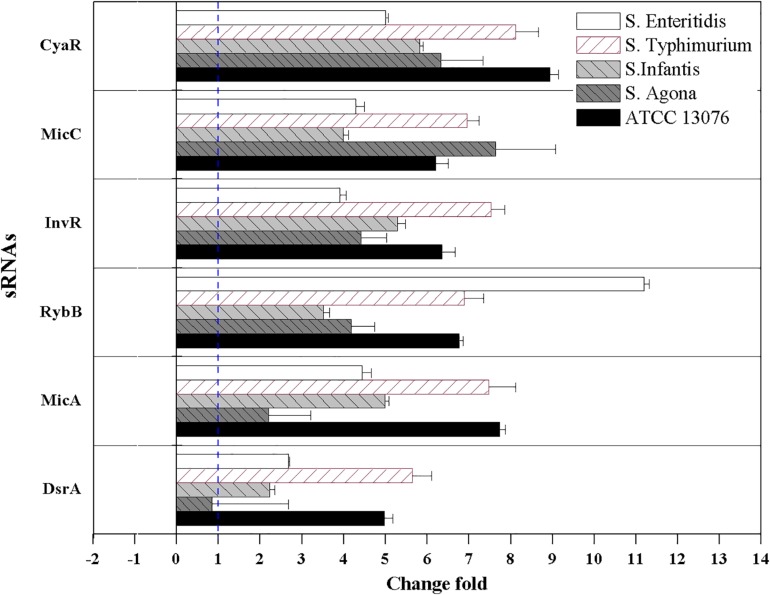
The relative expression of sRNAs of *Salmonella* strains when exposed to acidic stress. Each symbol indicates the mean ± standard deviation of five independent experiments (*n* = 5).

The relative expression levels of six sRNAs in *Salmonella* strains exposed to the three stresses for 3 h were measured using reverse transcription quantitative PCR combined with the 2^–ΔΔCt^ method. For acidic stress, the relative expression levels of sRNAs are shown in Figure 4. The expression of *RybB* in *S.* Enteritidis was highest, with a relative expression level of 11-fold compared with that of the untreated control. Except for *DsrA* in *S*. Agona, the other sRNAs showed significant upregulation (log_2_[2^–ΔΔCt^] > 1).

**FIGURE 5:**
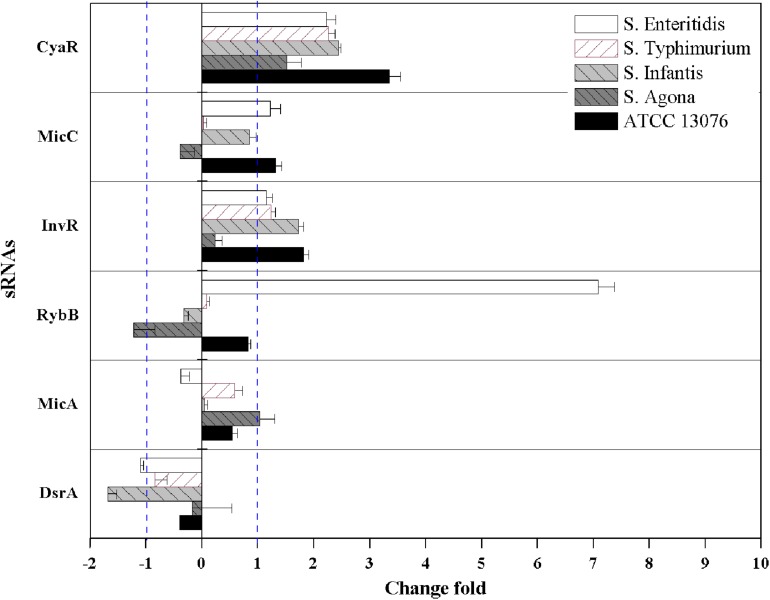
The relative expression of sRNAs of five *Salmonella* strains when exposed to hyperosmotic stress. Each symbol indicates the mean ± standard deviation of five independent experiments (*n* = 5).

For hyperosmotic stress, the relative expression levels of six sRNAs are shown in Figure 5; dramatic variations were observed in the relative levels of the sRNAs. The fold change in *RybB* in *S.* Enteritidis was significantly higher (sevenfold) than that in other strains; for example, the lowest expression (−1.3-fold) was observed in *S.* Agona. For the sRNA *CyaR*, higher relative expression was observed in all tested strains, with log_2_(2^–ΔΔCt^) values of greater than 1. The fold expression of InvR was higher, except for in *S.* Agona. Compared with the sRNAs described above, the fold expression of *DsrA* was decreased for all *Salmonella* strains, particularly for *S.* Infantis. No significant differences in *MicA* expression were observed.

**FIGURE 6:**
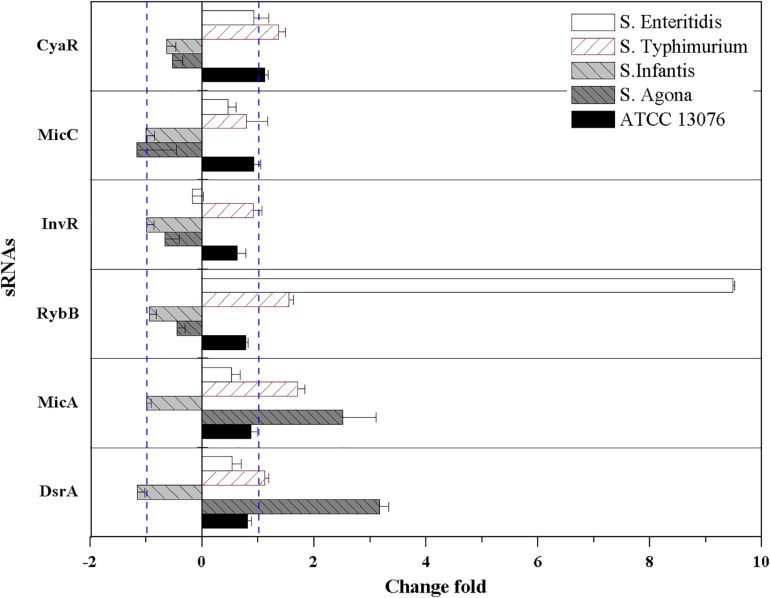
The relative expression of sRNAs of five *Salmonella* strains when exposed to oxidative stress. Each symbol indicates the mean ± standard deviation of five independent experiments (*n* = 5).

For oxidative stress in the tested *Salmonella* strains, relative expression levels of sRNAs are shown in Figure 6. Compared with the acidic environment, the relative expression levels of sRNAs in *Salmonella* strains were not significantly higher after exposure to an oxidative environment. The expression of *RybB* was highest in *S.* Enteritidis, showing a relative expression level of 9.5-fold that of the untreated control. For *S.* Typhimurium, the relative expression levels of *CyaR*, *RybB*, and *MicA* increased, and similar trends were observed for *MicA* and *DsrA* of *S.* Agona (log_2_[2^–ΔΔCt^] > 1). However, the relative expression levels of all tested sRNAs for *S.* Infantis were lower (log_2_[2^–ΔΔCt^] < 0).

## Discussion

As a widespread foodborne pathogen, *Salmonella* sp., including more than 2500 *Salmonella* serovars, prefer various habitats related to animal or human infection, resulting in 122 outbreaks, 3061 illnesses, and several food recalls in the U.S. in 2017 (Centers for Disease Control and Prevention [CDC], 2019). Controlling *Salmonella* growth and survival is essential for ensuring food safety. During the processing, storage, and distribution of food, bacteria usually encounter various environmental stresses (Hahm and Bhunia, 2006). For example, some foods, including dry-cured meats and sauces, have relatively higher salt contents (even more than 8.0%), forming a relative hyperosmotic environment and promoting bacterial survival. Additionally, juices, which have lower pH values, can provide an acidic environment. Chlorine-based disinfectants (chlorine levels commonly ranged from 50 to 200 ppm) and electrolyzed water with concentrations from 50 to 500 ppm are commonly applied to remove bacterial cells, inducing an oxidative environment (Bridier et al., 2015). In modern food processing plants, several sanitizers are often combined to detach biofilms formed by pathogens, which can induce multiple stresses (Galiè et al., 2018; González-Rivas et al., 2018). There is large heterogeneity and diversity in responses to environmental stresses among bacterial isolates; thus, it is essential to explore the cell survival profiles of bacteria in the presence of specific environmental stresses.

In the current study, five tested *Salmonella* strains exhibited great differences in resistance to acidic stress, with *S.* Enteritidis showing the highest tolerance. Consistent with these findings, recent evidence has shown that the serotype influences the acid tolerance and recovery of *Salmonella* (Richardson et al., 2018). Similar results were also reported by Cox et al. (2015), who demonstrated that the death of *Salmonella* cells exposed to acidic conditions depended on the serotype. The varying acidic resistance of the tested strains may have been related to diversity in the induction of specific proteins, contributing to the maintenance of pH or to the repair of damaged proteins and DNA, such as *RpoS* and *OmpR*; diversity in the proportions of saturated, unsaturated, and cyclic fatty acids; or diversity in post-translational modifications in regulatory proteins induced by stress, such as acetylation/deacetylation (Ren et al., 2015). The resistance of cells to acidic stress is concerning, particularly for injured but not dead bacterial cells, which could exhibit disruption of regular recovery. In this study, a major reduction in cell numbers after 1 h of exposure to stress was observed; this effect could be explained in part by the membrane fluidity induced by low pH and disruption of the liquid-crystalline balance (Yang et al., 2014). Similar results were reported by Chakraborty et al. (2017) using single-cell analysis; in their study, the intracellular pH reached the threshold of 6.5 after the cells were subjected to acid stress for 90 min. It was concluded that high-intensity acid stresses may rapidly reduce the internal pH below this threshold, increasing OmpR dimerization and DNA binding.

sRNAs have been shown to be involved in the regulation of environmental stress response pathways, facilitating cell switch from sensitive to resistance lifestyle to rapidly adapt the challenging environments. All six tested sRNAs in this study were upregulated after exposure to acidic stress, with *RybB* in *S*. Enteritidis showing the highest fold change. *RybB* is typically activated by the envelope stress sigma factor, whose activity sharply increases under various stress conditions, such as acid and hyperosmotic stresses. Similar to *RybB*, *MicC* can also downregulate the synthesis of the major porin *OmpD* via Hfq-dependent base pairing (Fröhlich et al., 2011). Notably, in this study, the elevated expression of *MicC* was observed. The sRNA *CyaR* is regulated by the cAMP/CRP complex, and showed high expression levels in this present study. This sRNA is known to suppress levels of OmpX, which is involved in the response to acidic stress (Johansen et al., 2008), and its higher expression may inhibit the synthesis of outer membrane proteins to control substances entering the cell and facilitate resistance to the external acidic environment.

Five *Salmonella* strains displayed varying responses to hyperosmotic stress in this study. *S.* Enteritidis showed the smallest variation in cell number after exposure to hyperosmotic stress for 3 h. *S.* Enteritidis can survive in a hyperosmotic environment, which is unfavorable for food storage and preservation. Bacterial cells exposed to high salt stress experience an instantaneous efflux of water out of the cell, which leads to the shrinkage of the cytoplasmic volume, decreased inflation pressure, and increased concentrations of intracellular metabolites. Filamentation or elongation of *Salmonella* may occur under stress induced by 5% NaCl (w/v) (Mattick et al., 2000). This treatment can also lead to impairment of essential cellular functions, thereby resulting in bacterial cell death (Smet et al., 2015; Burgess et al., 2016). However, bacterial cells can also react to variations in hyperosmotic stress in different ways to adapt to the environment (O’Byrne and Booth, 2002; Noriega et al., 2014). The *S.* Enteritidis strain used in this study showed a good ability to resist stress induced by 8% NaCl. Further studies are needed to examine this result in more detail, particularly for analysis of high-salt foods, such as sauces, peanut butter, and seafood. Indeed, these food systems exhibit high potential for *Salmonella* contamination, and cells that can resist the stress and survive for long periods increase the overall risk to consumers.

The sRNAs *CyaR* and *InvR* showed upregulation in all tested strains exposed to hyperosmotic stress. Previous studies have shown that *CyaR* is positively regulated by sigmar E (σ^E^) and the global regulator Crp but promotes the decay of *ompX* mRNA (encoding a major outer membrane protein), which is involved in the response to environmental stress in *Salmonella* (Johansen et al., 2008; De Lay and Gottesman, 2009). However, *CyaR* has been shown to exhibit decreased expression under multiple chemical stress conditions (Rau et al., 2015), and differences in the applied patterns of stresses (e.g., salt versus trace elements and vitamins) could explain such discrepancies. *InvR* is an Hfq-dependent sRNA that post-transcriptionally suppresses the synthesis of the abundant outer membrane protein OmpD (Pfeiffer et al., 2007), which is involved in stress adaptation in microorganisms.

Great diversity in oxidative resistance was observed in this study, with *S.* ATCC 13076 displaying the highest tolerance. Consistent with this finding, diversity in growth curves of *S*. Typhimurium, *S*. Heidelberg, and *S*. Enteritidis under chlorine-induced oxidative stress was observed by Dhakal et al. (2018). Similarly, survival rates were substantially increased among 16 strains of bacteria response to oxidative stress after 10 min of incubation (Gomes et al., 2018). The variety of metabolic states for each serotype under oxidative stress could explain such discrepancies. Indeed, Liu et al. (2017) demonstrated that reducing nucleotide and amino acid biosynthesis, suppressing energy-associated metabolism, and promoting fatty acid metabolism were involved in the bacterial response to oxidative stress. A possible link between the stress response and outer membrane proteins may also partly explain the variety of oxidative stresses. For example, a previous study demonstrated the multifunctional characteristics of the outer membrane protein TolC in oxidative stress resistance (Lee et al., 2016). These findings supported the conclusion that the behaviors of bacterial isolates under specific stresses may be strain-/serotype-dependent and may not be related to other factors, such as the source of isolation. In addition to the sRNA *RybB* in *S*. Enteritidis, *MicA* and *DsrA* in *S*. Agona showed high expression levels in this study. *DsrA* promotes the expression of *DksA*, which can increase the availability and stabilization of the core RNA polymerase for binding to sigma S, a global master regulator affecting more than 500 stationary phase and stress-induced genes. Additionally, *DksA* also contributes to the switch in the transcription program needed for stress adaptation (Girard et al., 2018). Moreover, overexpression of *MicA* directly induces the production of OmpC-enriched outer membrane vesicles, which protect against various stresses (Choi et al., 2017).

## Conclusion

In this study, the variations in the responses of *Salmonella* isolates to acidic, oxidative, and hyperosmotic stresses, according to the reduction numbers of bacteria subjected to stresses, were determined. The differences in the relative expression levels of sRNAs were also accessed, such as *RybB*, *CyaR*, and *DsrA*, involved in the regulation of outer membrane proteins and sigma factor under specific stresses. Based on these findings, it was concluded that the behaviors of *Salmonella* isolates under specific stresses were strain-dependent and not likely to be related to other factors, such as the source of isolation. Further studies with more serotypes and isolates from various sources should be tested and confirm these findings. These results provide insights into the resistance profiles of food-related strains, yielding meaningful information regarding the control of *Salmonella* in the food industry.

## Data Availability Statement

All datasets generated and analyzed for this study are included in the article/supplementary material.

## Author Contributions

HW, XX, and GZ designed the study. HW, MH, XZ, and BP performed the methodology. HW and MH wrote the first draft of the manuscript. All authors contributed to writing the original draft, review, and editing the manuscript.

## Conflict of Interest

The authors declare that the research was conducted in the absence of any commercial or financial relationships that could be construed as a potential conflict of interest.
